# How to Integrate HIV and Sexual and Reproductive Health Services in Namibia, the Epako Clinic Case Study

**DOI:** 10.5334/ijic.2488

**Published:** 2017-07-12

**Authors:** Tomas Zapata, Norbert Forster, Pedro Campuzano, Rejoice Kambapani, Heena Brahmbhatt, Grace Hidinua, Mohamed Turay, Simon Kimathi Ikandi, Leonard Kabongo, Farai Zariro

**Affiliations:** 1 World Health Organization (WHO), NA; 2 Ministry of Health and Social Services (MoHSS), NA; 3 United Nations Population Fund (UNFPA), NA; 4 Joint United Nations Programme on HIV/AIDS (UNAIDS), NA

**Keywords:** Health service integration, integration, HIV/SRH integration, people-centred integrated care, person-centred integrated care, longitudinality, models of service integration

## Abstract

**Introduction:**

During the past two decades, HIV and Sexual and Reproductive Health services in Namibia have been provided in silos, with high fragmentation. As a consequence of this, quality and efficiency of services in Primary Health Care has been compromised.

**Methods:**

We conducted an operational research (observational pre-post study) in a public health facility in Namibia. A health facility assessment was conducted before and after the integration of health services. A person-centred integrated model was implemented to integrate all health services provided at the health facility in addition to HIV and Sexual and Reproductive Health services. Comprehensive services are provided by each health worker to the same patients over time (longitudinality), on a daily basis (accessibility) and with a good external referral system (coordination). Prevalence rates of time flows and productivity were done.

**Results:**

Integrated services improved accessibility, stigma and quality of antenatal care services by improving the provider-patient communication, reducing the time that patients stay in the clinic in 16% and reducing the waiting times in 14%. In addition, nurse productivity improved 85% and the expected time in the health facility was reduced 24% without compromising the uptake of TB, HIV, outpatient, antenatal care or first visit family planning services. Given the success on many indicators resulting from integration of services, the goal of this paper was to describe “how” health services have been integrated, the “process” followed and presenting some “results” from the integrated clinic.

**Conclusions:**

Our study shows that HIV and SRH services can be effectively integrated by following the person-centred integrated model. Based on the Namibian experience on “how” to integrate health services and the “process” to achieve it, other African countries can replicate the model to move away from the silo approach and contribute to the achievement of Universal Health Coverage.

## Introduction

### Background

Most African countries adopted a Primary Health Care (PHC) approach after Alma Atta Declaration in 1978 [1]. However, in the 80’s a selective PHC was encouraged with a focus on specific high impact interventions [2]. In the 90’s because of the HIV emergency and the earmarked donor funding, a “vertical” approach to provide health services was implemented [2,3]. This has created a dual system in many African countries, whereby the HIV services have the adequate number of human resources, the right infrastructure and are providing quality HIV services, while Primary Health Services (including Sexual and Reproductive Health Services –SRH) are understaffed, have old infrastructure and provide poor quality health services [4].

The HIV/SRH Integration agenda has been gaining global momentum since June 2004 when UNFPA and UNAIDS signed the New York Call to Commitment on Linking HIV/AIDS and Sexual and Reproductive Health [5,6]. Integration of both services according to the WHO definition of service delivery integration [7], ensures people receive a continuum of care according to their needs throughout their life course. The recently developed WHO Framework on “integrated people-centred health services” also opens a window of opportunity to integrate health services [8].

One of the initiatives to improve service delivery Integration is the UNFPA/UNAIDS “HIV/SRH Integration project” that has been conducted in seven Southern African countries, including Namibia [9]. Epako clinic is one of the seven pilot health facilities in Namibia implementing the new person-centred integrated model. Epako clinic was selected as a case study because of the high level of integration of services and the high level of commitment by the clinic staff and regional management with the new integrated model.

### Problem Statement

Namibia has a generalized, mature HIV epidemic with a HIV prevalence of 14% [10] and high antiretroviral (ARV) coverage (90%).

From a SRH perspective, there is high coverage of Antenatal Care (97%), skilled deliveries (88%) and prevalence of modern contraceptive use (50%). However, the country continues to experience unacceptably high levels of maternal mortality (200/100,000), teenage pregnancy (18.6%) and unmet need of Family Planning (FP) (15%) [10,11].

HIV and SRH services in Namibia are provided in silos, with limited integration at service delivery. Beyond poor SRH delivery and impact indicators, the diminishing external funding for the HIV response, due to the new classification of Namibia as an upper-middle income country [12] also justifies an HIV/SRH integration strategy to improve sustainability.

Analysis from the “pre” baseline assessment showed that health services in the Epako clinic were provided following a vertical, non-integrated approach and operated as two different clinics with very limited interaction and coordination between the “PHC” and the “ARV” clinics. PHC services were also organized by diseases (or programmes) with different consulting rooms for different “health programmes”. Not all the PHC services were provided on a daily basis. Thus this posed a challenge for accessibility, since many patients came from remote rural farms incurring high transport costs for each visit to the healthcare system. As a consequence, this also increased loss in patients’ follow up.

In view of the above situation in Epako clinic and in the other six pilot health facilities in Namibia there was a need of re-thinking and redefining the service provision model in Epako clinic to provide better integrated services and improve quality of services.

Integration is understood in many different ways [13,14] and there is not much evidence available internationally on how better integrate vertical programmes, the process to follow to integrate them and the benefits of doing so.

The main objectives of this paper are to describe “how” HIV and SRH services were integrated in Epako clinic, the “process” to reorganize health services and present some “results” of the new person-centred integrated model from Epako clinic.

## Methodology

**Figure 1:**
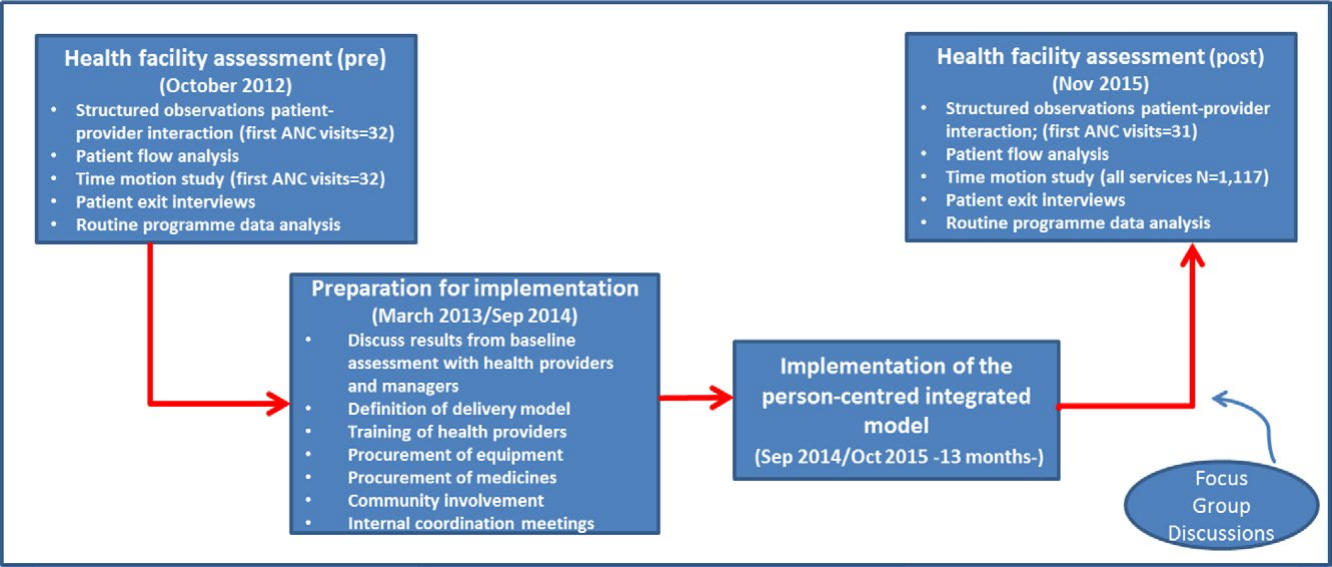
Phases of the study.

We conducted an observational pre-post study on 7 pilot health facilities in Namibia. This paper describes the findings from the Epako clinic. Figure 1 outlines the framework used for our study.

A health facility baseline assessment was conducted in October 2012 that included: 1) structured observations of patient provider interactions; 2) patient flow analysis; 3) time motion study; 4) patient exit interviews and 5) routine programme data analysis. Structured observations were conducted by the research team. Each researcher followed one nurse and directly observed the nurse-patient interaction without interfering. A checklist was used to assess quality components of the services provided and also general qualitative observations were captured. In total 32 first antenatal care interactions, 13 antenatal care follow up visits, 5 postnatal care visits, 25 outpatients visits and 15 HIV treatment consultations were observed.

**Figure 2:**
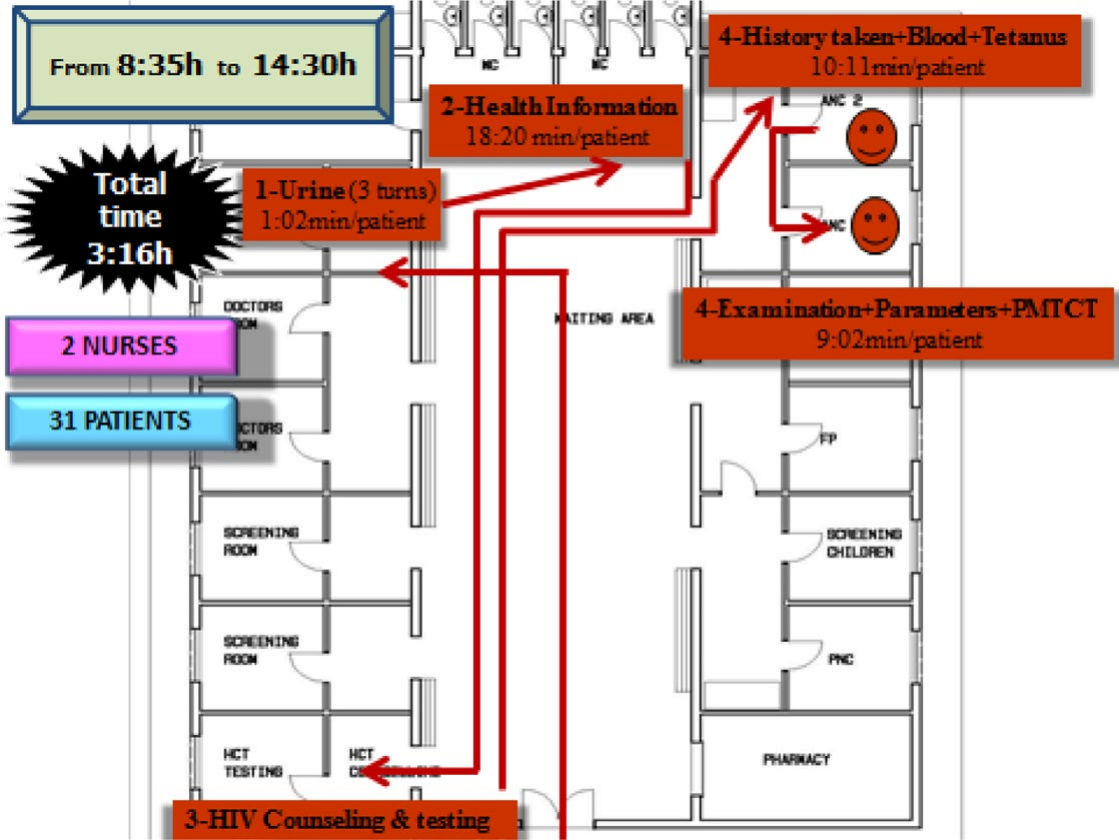
Time motion first antenatal care patients in Epako clinic (health facility baseline assessment).

Patient flow analysis and time motion study were conducted by tracking times of all 32 first antenatal care patients attending the clinic during one week. Each patient was given a card where the research team (located inside and outside the consulting rooms) recorded the type of service provided, the name of the consulting room, the category and name of the health provider and the start and finish time for each service and waiting times. Data obtained were plotted on a map of the health facility allowing us to track patient flow and time spent in each area (Figure 2).

Patient exit interviews were conducted on all first antenatal care patients (n = 32) seen in one week. Patients were asked about the total expected time to get first antenatal care services (before coming to the clinic), time and cost to reach the clinic and overall satisfaction. Satisfaction with the service received ranged from: “very good”, “good”, “poor”, “very poor”. 4 points were allocated to “very good” answers, 3 to “good” answers, 2 to “poor” answers and 1 to “very poor” answers.

Routine programme data analysis of selected indicators was collected from the District Health Information System to define the baseline.

In addition to the health facility assessment, focus groups discussions were conducted with nurses and patients in November 2014, two months post implementation of the people-centred integrated model.

Epako is one of the seven pilot health facilities included in our study. It is a public town clinic in the Omaheke region of Namibia, with a catchment area of 85,000 km^2^ and catchment population of 21,000 people. Most people live in rural areas (70%). HIV prevalence among pregnant women is 12.7%. Epako follows about 4,500 patients living with HIV from the entire Omaheke region. A majority of HIV infected individuals (64%) are on ARV treatment. The workload indicators and staffing needs (WISN) ratio which provides an indicator of the clinic work load and the number of staff needed is 0.95. This indicates that Epako had the right number of health workers to cope with the existing workload in the clinic.

Based on the findings of the health facility baseline assessment, a person-centred integrated service delivery model (described below) was conceptualized and preparations for the implementation of the new integrated model were initiated in the Epako clinic. A participatory approach was followed to define the model and to agree on how to rearrange health services at the clinic.

Health workers from Epako clinic were trained in those areas of clinical practice where they had some gaps (HIV treatment, TB treatment, family planning, etc.). In service training was encouraged and when it was not possible health workers were sent to formal trainings provided by Ministry of Health and Social Services in other locations.

The person-centred integrated model was implemented at the Epako clinic in 2014 and a post integration assessment was conducted about 13 months later in 2015.

The post integration assessment followed the same methodology as the baseline assessment (structured observations, patient flow analysis, time motion study, patient exit interviews and programme data analysis), except that time motion data were captured from all patients visiting the clinic during one week (not only first antenatal care patients). In total 1,207 patients visited Epako clinic during the week of the post integration assessment and we have complete data on 1,117 patients (93%) as in about 7% of the patients, forms were not adequately completed or lost.

Times from all 31 first antenatal care patients seen during that week were also captured.

Indicators from the pre-health facility baseline assessment were compared with indicators from the post-health facility assessment. Data from different databases (DHIS, EDT, TB and EPMDS) from Ministry of Health and Social Services were used to analyse the uptake of services and the impact of some interventions.

## Description Of The Care Practice

### The old service delivery model in Epako clinic (before integrating health services)

As described in the introduction the “pre” health facility baseline assessment showed that health services in Epako clinic were very fragmented, vertically organized. In fact it operated as two separate clinics: the “Epako HIV clinic” and the “Epako Primary Health Care clinic”. In the “Epako Primary Health Care clinic” services were also provided in silos, specific health services were provided in specific rooms during specific days of the week and by specific nurses. Therefore patients had to be internally referred from one consulting room to another (from one nurse to another) to get all health services needed (eg from family planning room to outpatient room) and in some cases they had to come on different days of the week to get all services. In the Epako HIV clinic, HIV treatment was provided by three nurses, one doctor and two community counsellors on a daily basis. Coordination between the two Epako clinics was very limited. This vertical way of organizing health services was the standard for Namibia.

### The new person-centred integrated model

Based on the results of the “pre” baseline assessment, a new model of service delivery integration was developed for the Epako clinic by WHO, UNFPA and UNAIDS in collaboration with Ministry of Health and Social Services (MoHSS). This model follows the four main pillars of Primary Care (Accessibility, Comprehensiveness, Longitudinality and Coordination) [15].

Under the new integrated, “person-centred” model, health services in the Epako clinic were provided from Monday to Friday (accessibility), by the same nurse following up the same patients over time (longitudinality), providing a range of services based on patient’s needs and always in the same consulting room (comprehensiveness).

This “patient-centred” model entails that patients are allocated to one nurse who will always follow them up in the same consulting room. When patients come to the clinic, they first go to “the reception” where the clerk will allocate them to one of the nurses in the clinic and will write the correspondent number of the nurse in the health passport card of the patient. The allocation process is done according to the two following criteria: 1) Language. All patients should be allocated to a nurse speaking the same language and 2) Workload. Allocation of patients only takes place the first time that patients are coming to the clinic under the new model. Once the patient has been assigned a number (a nurse) in their health passport card, they will always go to the same nurse who always stays in the same consulting room. To ensure that all nurses share an equal patient burden, patients that require more time to be attended to are equally allocated among the different nurses (only during the first time patients are allocated to one nurse). For example, the first antenatal care visit normally takes from 30 to 45 minutes, thus first antenatal care patients are equally allocated to different nurses.

Each nurse has her/his own consulting room with its own waiting area. This allows patients to know where to wait and when they are going to be seen by the nurse. At the same time, it allows nurses to see the number of waiting patients and identify if one of them requires to be seen first.

Each consulting room is fully equipped with all needed equipment (scale, blood pressure machine, needles, syringes, stretcher, dressing equipment, etc). Each room also has all the monitoring and evaluation registers that need to be completed (antenatal care, family planning, outpatient, HIV, etc) and all the essential medicines provided in Epako clinic (antibiotics, pain killers, eye drops, etc). Only antiretroviral medicines are not provided in the consulting room since they are provided in the HIV pharmacy. The reason for having a HIV pharmacy is that it has a patient base ART electronic database (EDT) to monitor each patient.

Services provided in the Epako clinic include: adult and child “screening” (in Namibia it refers to outpatient visits for common signs and symptoms such as fever, diarrhoea, headache, abdominal pain, injuries, etc), immunisation, nutrition monitoring, family planning, antenatal care, postnatal care, HIV counselling and testing, HIV treatment, TB treatment, pap-smear, and wound dressing. Only HIV counselling and testing is not provided by the nurse, so the patient is internally referred to the community counsellor within the clinic to do the pre-test counselling and the testing. Once the patient has been tested he is referred back to his/her nurse. The reason is that Epako clinic has community counsellors specifically trained for HIV counselling and testing. However, in the future we would also like to move towards provider initiated counselling and testing (PITC) done by the nurse.

In some complicated cases, nurses may need to internally refer the patient to the medical doctor available in the clinic. If the doctor is not in the clinic, the patient will be referred to the District hospital that is 5 Km away from Epako clinic.

### Preparation for the Implementation of the new model and implementation

The preparation for the implementation of the new integrated model took place in different steps described in Table 1. A participatory approach was followed in most of the steps described in the table. Health workers and managers were engaged since the beginning of the operational research and were part of the process of reorganizing health services in their health facilities. Training and equipment needs were identified with them and they were empowered to prepare the health facilities to start the new person-centred integrated model. This step took 18 months due to the participative approach followed and due to the long delay in the delivery of the needed medical equipment.

**Table 1:** Steps followed to prepare for the implementation of the new person-centred integrated model of health service delivery in Epako Clinic, Namibia.


***Step 1.*** To inform and involve the Omaheke Regional office, the Gobabis Health District and the management in Epako clinic in conducting the study to create some buy in.
***Step 2.*** To show the results from the assessment to Epako’s health workers, managers from Gobabis health district and Omaheke Region. Presentation of results raised awareness among health staff and managers about the problems and key bottlenecks on how they were providing health services. At the same time, a better understanding and interest of the new integrated model was created.
***Step 3.*** To conduct a one day workshop with health workers from Epako clinic and some managers from the district and region on how to re-arrange health services in the clinic by following the new integrated model. New ideas and practical solutions were suggested by health staff from Epako on how to re-organize services. For example nurses decided the room they wanted to be in, which priority trainings were needed by each nurse, what equipment was needed and which medicines and M&E books were needed in each consulting room. After decisions were agreed on, a role play was performed to fully understand what the new model looked like.
***Step 4.*** Training health staff on the required priority trainings. Some trainings took place outside the clinic, such as the Integrated Management of Adolescent and Adult Illness training and others within the clinic (in service training). In this case, experienced nurses taught other nurses how to perform, for example, a pap-smear, how to complete the HIV M&E register book, how to explore a ANC patient, etc. All these skills were learnt by nurses during their nursing studies but by serving only certain specialized care it made necessary to refresh them on these areas.
***Step 5.*** To get the minimum new equipment needed in each consultation room.
***Step 6.*** To undertake internal preparations within the clinic before starting the new integrated model. Internal meetings were organized to discuss how some specific aspects or problems could be sorted out. For example how to collect drugs from the pharmacy to the consulting room, how to share some vaccines that can only be reconstitute for one day, how to deal with TB patients, how to transport HIV records without compromising confidentiality, etc. Epako health workers took the lead at this stage and showed a high level of commitment, motivation and knowledge about the new integrated model.
***Step 7.*** To involve the community. Church pastors and community leaders were briefed about the new model and they transmitted the information to the communities. This was proved to be a very effective way of reaching the community.
***Step 8.*** To start the provision of services following the new system. During the first two weeks UNFPA, UNAIDS, WHO and MoHSS provided technical support within the clinic. New adjustments were made based on new challenges identified. Nurses supported each other by sharing technical knowledge and skills with their colleagues. It was frequent to see a nurse asking another nurse on how best to do something. At the end of the day these challenges and positive experiences were shared in “team” meetings where all nurses felt free to express themselves and share their experiences and solutions.


Implementation of the new person-centred integrated model started in September 2014 and continued until October 2015 (13 months), just before the post health facility assessment was conducted in November 2015. After the post assessment Epako clinic has continued providing integrated health services.

## Results

### Organization of Health Services

Results from the health facility baseline assessment showed that not all services were provided on a daily basis in Epako clinic. First antenatal care was provided only once a week (Tuesdays) and antenatal care follow up was provided three times per week (Monday, Wednesday and Thursday) (Table 2).

**Table 2:** Health services provided per day, by health provider and by room in Epako clinic (pre-baseline assessment).

Health services	Mon	Tue	Wed	Thu	Fri	Where	Health provider

**First antenatal care**		X				Antenatal care room	Nurse 1
**Antenatal care follow up**	X		X	X		Antenatal care room	Nurse 1
**Postnatal care**	X	X	X	X	X	Immunisation room	Nurse 2
**Immunization**	X	X	X	X	X	Immunisation room	Nurse 2
**Family planning**	X	X	X	X	X	Family planning room	Nurse 3
**Outpatients (adults)**	X	X	X	X	X	Adult outpatients room	Nurse 4
**Outpatients (children)**	X	X	X	X	X	Child outpatient room	Nurse 5
**HIV counseling and testing**	X	X	X	X	X	HCT room	2 community counselors
**Antiretroviral provision**	X	X	X	X	X	HIV clinic	3 nurses + 1 medical doctor + 2 community counselors
**Tuberculosis**	X	X	X	X	X	TB room	Nurse 6
**Dressing**	X	X	X	X	X	Dressing room	Nurse 7
**Pap smear**	X	X	X	X	X	Antenatal care room	Nurse 1

42.5% of first antenatal care patients went to Epako clinic on a different day than a Tuesday. Therefore they could not get the service they needed and they were told to come back on the next Tuesday.

During the focus group discussions, patients described that before integration, HIV positive patients had to go to the “Epako ARV clinic” and as a consequence of this, everyone in the community knew who was HIV positive. This led to many of the participants feeling stigmatised by attending the ARV clinic.

1,207 patients visited Epako clinic during the post-integration assessment and data was captured from 1,117 patients. Out of the 1,117 patients, 28% attended “adult outpatient services” (prevention and treatment of clinical conditions), 2.8% demanded first antenatal care services, first visit for antiretrovirals was demanded by 4 patients and HIV antiretroviral follow up was demanded by 192 patients.

Results from the “post” health facility assessment, after the implementation of the new integrated model, showed that all health services in Epako clinic were provided on a daily basis and 0% of first antenatal care patients (n = 31) were told to come back on another day. Data from the exit interviews showed that the average time for antenatal care patients to reach the clinic was 41 minutes and the average cost was 8.71 Namibian Dollars (0.5 USD).

By providing all services (including HIV services) by the same nurse in the same room, community members could not know who was HIV positive. Qualitative data from focus groups discussions showed that stigma of HIV patients was minimized with the new integrated patient-centred model.

### Time motion analysis (Table 3)

Table 3 summarizes results from the time motion analysis of first antenatal care patients (n = 32 in the “pre” baseline assessment and n = 31 in the “post” assessment). As can be seen the total time spent in the clinic for the first antenatal care visit was reduced by 16.4% (p < 0.05) after integrating services.

**Table 3:** Time motion indicators, nurse productivity and satisfaction for first antenatal care (ANC) in Epako clinic (times expressed in hour:min).

Indicator	Before implementation of integrated model	After starting implementing the integrated model	Difference	% change

**Total time in the clinic by first ANC patient**	03:05	02:35	00:30	–16.4%*
**Time inside the first ANC room by patient**	00:36	00:20	00:16	–45.4%**
**Time inside the HCT room by first ANC patient**	00:25	00:29	–00:04	16.0%*
**Time in the waiting area by first ANC patient**	02:03	01:46	00:17	–14.4%NS
**Expected time to be at the clinic by first ANC patient**	02:48	02:08	00:40	–23.9%*
**Nurse productivity in first ANC (# patients per nurse per hour)**	1.6	3.0	1.4	85.2%***
**Time to reach the clinic by first ANC patient**	No data	00:41	N/A	N/A
**Satisfaction by first ANC patient (4 Very good; 3 Good; 2 Poor; 1 Very poor)**	3.20	3.13	–0.07	–2.2%NS

*p<0.05; **p<0.001; ***p<0.001; NS-Not significant.

The time first antenatal care patients were inside the antenatal care consulting room to get the following services: parameters (blood pressure, urine, weight and braquial perimeter), health information, blood withdrawal, medical history record, tetanus vaccine, physical examination, sulphate ferrosus and prevention of mother to child transmission of HIV if needed, was reduced in 45.4% (p < 0.01) (from 36 to 20 minutes).

The total time that first antenatal care patients spent in the waiting area was reduced by 14.4% (from 2 h 03 min to 1 h 46 min). However, this result was not statistically significant. This could be explained by the fact that first antenatal care services were provided quite comprehensively before the new integrated model was implemented (96.6% coverage) [10]. One nurse was providing most of first antenatal care services at once in the same room. In other pilot clinics where first antenatal care services were more fragmented, the waiting and uptake of services gains were more significant [16].

### Patient’s expectations

Results from patient exit interviews show that the expected time to be at Epako clinic by first antenatal care patients was reduced in 23.9% (p < 0.05) after the implementation of the person-centred integrated model (Table 3).

### Health provider’s productivity

Nurses productivity was measured as the number of first antenatal care patients seen per nurse per hour. It improved from 1.6 in the “pre” assessment to 3.0 after the implementation of the new integrated model. This implies an 85.2% (p < 0.001) increase in nurses’ productivity with the new integrated model.

### Patient’s satisfaction

The average score in the “pre” health facility assessment was 3.20, meanwhile after the implementation of the integrated model was 3.13. Since this difference is not statistically significant we cannot conclude that satisfaction has changed with the new integrated model.

### Utilization of health services (Table 4)

Results from this section have been taken from the District Health Information System (DHIS), TB, EDT and EPMDS databases from Ministry of Health and Social Services for Epako clinic. The period of analysis of data before the implementation of the integrated model is from January 2012 until 31 August 2014. Data for the analysis of the implementation of the integrated model was taken from 1^st^ October 2014 to 31^st^ October 2015.

**Table 4:** Utilization of services (average per month) in Epako clinic before and after the implementation of the person-centered integrated model.

INDICATOR	Before implementation of integrated services (from Jan 2012 to Aug 2014)	After starting implementation of integrated services (from Oct 2014 to Oct 2015)	Balance	% change

**Average number of first ANC patients**	95.5	95.5	0.0	0.0%
**ANC follow-up visits**	335.3	177.9	–157.4	–46.9%
**Postnatal care visits**	71.5	60.0	–0.2	–16.0%
**Family planning first visits**	31.3	37.4	6.1	19.5%
**Family planning follow-up visits**	635.6	571.0	–64.6	–10.2%
**Under 5 screening first visits**	394.2	349.1	–0.1	–11.4%
**>18 years screening first visits**	784.1	696.5	–0.1	–11.2%
**Total screening re-visits, follow-up**	481.7	1007.1	525.4	109.1%
**Pap Smear per month**	18.6	13.5	–5.2	–27.7%
**New malnutrition cases**	10.0	10.4	0.4	3.8%
TB treatment cure rate*	96.3%	97.0%	0.7%	0.7%
**TB treatment success rate**	96.3%	97.0%	0.7%	0.7%
**New HIV patients on antiretrovirals**	27.1	24	–3.1	–11.4%
**Routine refills of antiretrovirals**	654.3	761.1	106.8	16.3%
**HIV patients who stopped ARV treatment**	6.9	6.4	–0.5	–7.2%
**HIV patients who deceased**	15	12	–3.0	–20.0%

* Reported every tremester (93.3% refers to the average rate of the 1st, 2nd & 3rd Trimester of 2014). 97.0% is for the 4th trimester 2014. This indicator is only possible to be collected after 12 months.

Table 4 summarizes the utilization of health services in Epako clinic before and after implementing the person-centred integrated model. The average number of first antenatal care patients per month remained constant (n = 95.5). The average number of antenatal care follow-up visits per month reduced by 47%, the average number of postnatal care visits decreased by 16%, the average number of family planning first visits increased by 19.5%, the average number of family planning follow-up visits per month decreased by 10.2% and the average number of pap smear performed per month decreased by 27.7%.

The significant decrease in the number of antenatal care follow-up visits (47%) was driven by a change in policy regarding antenatal care visits at the national level. Guidelines from Ministry of Health and Social Services used to set a high number of follow-up visits for antenatal care patients (eight), however recently, a new circular has been distributed to implement “focused antenatal care”, which aims to provide only four visits for antenatal care. Therefore, most of the health facilities in the country have experienced significant reductions in the number of antenatal care follow-up visits during the past 18 months.

The average number of under-5 screening visits per month decreased by 11.4%, and the average number of screening visits of patients 18 years old or older decreased by 11.2%. The average number of malnutrition diagnosis per month increased by 3.8%. However, the total number of screening re-visits or follow-up visits increased by 109.1%. In addition, the TB treatment cure rate increase moderately with the person-centre integrated model from 96.3% during the first three trimester of 2014 to 97.0% in the last trimester of 2014 (0.7%).

The average number of new HIV patients on antiretroviral per month decreased 11.4%. The average number of patients collecting timely routine refills of antiretroviral per month increased 16.3%. The average number of HIV patients who stopped antiretroviral treatment (adherence) per month decreased 7.2%. It also decreased the average number of HIV patients who deceased per month in 20%.

### Focus groups discussions with nurses and patients

#### Comprehensive services

With the new integrated model, patients get all the services they need at once with the same nurse. The advantage of this is illustrated by the following comment of a 32 years old female *“…so I don’t have to go to another room to get FP…are you sure?…I can’t believe it. So now I can get everything I need at once!! This is like the private doctor, I don’t need to go to the private doctor any more”*. A mother could now get family planning and at the same time get treatment for her flu, grab some condoms and treat her sick child. The benefits of this integrated approach were shared by a nurse: *“Patients love coming with her baby and get family planning at the same time”*.

#### Longitudinality

Another advantage of the new model is that patients are always seen by the same nurse. *“This is my nurse”* illustrates how powerful it is building a trustful nurse-patient relationship. Patients now feel that someone concrete is taking care of them and cares about them and is familiar with their history and comprehensive health needs. At the same time, the nurses feel the responsibility of providing quality services and looking beyond the clinical problem *“You know all your patients” “By looking to your patients you know whether something is wrong. For example Maria came for a headache but I could see that there was something else, she didn’t look as usual, I asked her some questions and then she confessed me that her husband told her he was HIV+. She was scared of taking an HIV test”*.

Screening follow up has also been improved: *“You can follow up your patients. If your patient defaults you follow up, you know he/she didn’t came back. Before (previous way of providing services) you couldn’t know.”*

The new model has allowed patients to communicate in their linguistic mother tongue with their nurse since one of the main criteria to allocate patients to nurses is language. *“I never used to tell all my issues because I couldn’t speak the language of the nurse, now I can speak, now I can share my problems!!!”*. A nurse also reported that *“they love coming to the same nurse and being able to speak the same language”*.

Confidentiality can also potentially increase and HIV infected individuals no longer feel stigmatized. *“A lot of patients defaulted ARV treatment because everyone going to ARV clinic was known to be HIV+”; “now nobody knows why I am coming to Epako clinic, I feel much better”*.

Workload distribution can also improve with the new model. Most of the nurses in the clinic are happier now with the workload distribution. *“In the past some colleagues were relaxing and I was full of work, now we all have the same workload”*.

Another positive outcome is the sense of pride and ownership that nurses can achieve as demonstrated by the following comments *“Everyone wants to decorate their rooms. You have your own consulting room, you have your private consulting room with a smile”, “we nurses feel proud of having our own consulting room”*.

## Discussion

### Discussion of main findings

Our study shows that HIV and SRH services can be effectively integrated by following the person-centred integrated model implemented in Epako clinic. Integrated services improve accessibility and quality of antenatal care services by improving the provider-patient communication, reducing the time that patients stay in the clinic, reducing the waiting times and reducing the time expectations by patients. Epako integrated model also improves nurses productivity and minimizes stigma of HIV patients without compromising the uptake of TB, HIV, outpatient, antenatal care or first visit family planning services. This integration study highlighted the need for integration and the significant benefits to both the patients and health care providers.

Epako clinic is one of the seven pilot health facilities included in the pre/post observational study conducted in Namibia. Results from the seven pilot health facilities support the above findings [16].

Some authors may argue that the previous “vertical” way of organizing health services in Epako clinic has several advantages. These authors may argue that vertical organization of health services can assure delivery of health services and improve health outcomes in specific programmes such as HIV and TB [17], especially in countries with very weak health systems because of technical supervision, higher dedicated resources and direct monitoring and evaluation to ensure performance [18]. These authors oppose to integration of services because they consider that good quality of specific health services (HIV, TB, family planning) could be compromised by integrating them in general health services that does not function well and therefore could have a negative impact on utilization and on health outcomes [17,19]. Another argument to oppose to integration of health services is that it may reduce the financial allocation of resources to specific programmes [19].

However, results from our study show that by integrating health services following the “person-centred” model improves quality of HIV and SRH services by improving patient-provider communication, reducing total time in the clinic, reducing waiting times and by reducing time expectations. Results also show that nurses’ productivity almost doubled with the “person-centred” model. In addition, TB treatment cure rate slightly increased with the new model and several HIV indicators also improved (timely collection of ARVs, HIV+ patients who stopped ARV treatment and HIV+ patients who deceased per month). These results from our study challenge the above evidence against integration of services.

Our results are supported by some international evidence that claims that integrated services allows a more holistic approach to health, centred on the health needs of individuals [20] that delivery effective services [21,22] and allows for a more efficient use of scarce resources [19]. More specific evidence on HIV/SRH integration suggest that some benefits of integration includes improved access to HIV/SRH services, improve quality of services and enhance effectiveness and efficiency [14,23,24,25] and improve key outcomes [26].

There is a gap in the literature demonstrating effective integration models in resource poor settings, often leading to duplication of services [27]. Integration means many different things for different people [14], therefore integration delivery models also varies much. Different studies present different integration models, such us bidirectional integration [28], one stop shop [29], full integration. However there is not a clear framework to classify and describe all the different models of HIV/SRH integration.

The person-centred integrated model we have followed has some similarities with the so called “one-stop shop” model [27,29], but it adds the “comprehensiveness” and the “longitudinality” as critical characteristics.

The one-stop shop model does not necessarily entail that comprehensive health services are provided by one health provider within the same consulting room. Often services are provided by different providers in different rooms through internal referrals within the same health facility [29].

Longitudinality is within the concept of continuity of care, but it refers more specifically to the provision of services and follow up by the same health provider to the same patients over time [15,30,31]. The one-stop shop model provides all services within the same health facility, however, it does not necessarily imply that all services are provided by the same provider over time (longitudinality) [27]. The person-centred integrated model put the person at the centre of the system and provides the services they need at once by their “personal” health worker (longitudinality) speaking their same language, improving the quality of health services in the health care system by providing comprehensive services and holistic care.

The relevance of longitudinality is supported in our study by the substantial increase (109%) in the number of screening re-visits (follow-up) per month in Epako clinic. Nurses tend to do a better follow up of their patients if they are following the same patient over time and understand the patient’s history. Before the person-centre integrated model was implemented, nurses were rotating from one service to another and there was very limited follow up of patients seen in the outpatient room. On the other hand, there was a 10% reduction in the number of family planning follow up visits, a 27% reduction in the number of pap smear and a 16% reduction in postnatal care. This should highlight the importance of strengthening the booking system and the commitment by nurses to improve these figures.

Many countries in the world are delivering services following this “person-centred” model, which is based on the four main features of Primary Care [15,32]. Some of these countries include UK, Spain, Italy and Sweden. Evidence from these countries and others show that by providing accessible, comprehensive, longitudinal and coordinated primary care services improves effectiveness, efficiency, equity and quality of care [21,22,33,34,35,36,37]. However, there is a gap in the literature demonstrating effective integration models in resource poor settings. Our case study shows “how” to implement a “person-centred” integrated model in the context of a Southern-African middle-income country.

### Limitations

The data presented in this paper corresponds only to one health facility in Namibia where a person-centred integration approach was implemented. Therefore it may have limited external validity. However, one of the main purposes of this paper is to describe a case study on “how” to integrate HIV and sexual and reproductive health services and the “process” to do it. Hence the aim of the data presented here is to provide an example of what has been achieved in the particular context of the Epako clinic.

The person-centred integrated model requires all medical equipment and registers be available in each consulting room. This posed a challenge in Epako clinic since it took more than expected to procure the needed medical equipment.

The quality of the data from the District Health Information System (DHIS) from Ministry of Health and Social Services in Namibia is limited. It is a paper based system at the health facility level and uses aggregated data. Hence it should be taken cautiously.

Satisfaction results are of limited quality. Patients were asked when exiting the clinic the following question: “How was the service provided to you?” However, a bigger and better structured patient/provider satisfaction survey has been conducted in the pilot clinics after the implementation of the study and the findings will be available shortly.

### Implications and recommendations

There is some evidence in the literature showing the benefits of integration of HIV and sexual and reproductive health services, although the quality of evidence is seen as low [26,38] however there is limited evidence about the best model to follow to integrate services. The integration of sexual and reproductive health, TB, HIV prevention and treatment and other health services is critical in improving health outcomes and improving efficiency gains and provides an important pathway towards the 90-90-90 targets [39]. Future research should take time motion indicators for all services provided in the health facility. New health facilities in Namibia scaling up the person-centred integrated model are already following this approach.

Evidence from Epako clinic can inform Ministry of Health in Namibia to scale up integration of health services in other health facilities in the country. The patient centre integrated model can improve quality of care and contribute to the achievement of Universal Health Coverage in Namibia and in other African countries.

## Conclusion

Health services at the Epako clinic are provided in a unique way for a Public health facility in Namibia. As our health facility baseline assessment shows, health services in primary care in Namibia are fragmented, following a “vertical” approach, where patients are seen in terms of their “diseases” or “health programmes”. However, with the new integrated model, the focus is not on the disease but on the “person”. This “person-centred” model, considers people holistically and not a sum of his/her diseases.

The process of re-arranging health services from one model to another is not an easy one. Human resistance to change is natural and expected. However by following a participatory approach in Epako clinic, we were able to successfully train and implement a patient-centred model that empowered nurses and other health staff in the clinic to take their own decisions and decide how to apply the new model in the clinic.

Our study shows that HIV and SRH services can be effectively integrated by following the person-centred integrated model implemented in the Epako clinic. Integrated services improve accessibility and quality of antenatal care services by improving the provider-patient communication, reducing the time that patients stay in the clinic, reducing the waiting times and reducing the time expectations by patients. Epako integrated model also improves nurses productivity and minimizes stigma without compromising the uptake of TB, HIV, screening, antenatal care or first visit family planning services.

This person-centred integrated model and the process to implement it, has the potential to be rolled out to other clinics and health centres in Namibia and to other countries in the African Region and improving quality of health services, contributing to the achievement of Universal Health Coverage. If many countries in the world are putting “persons” at the centre, why should African countries not do the same?

## Reviewers

**Daniel Cobos Muñoz, MD MSc PhD**, Research fellow, Health Systems and Policies Research Group, Health Systems Research and Dynamical Modelling Unit, Epidemiology and Public Health Department, **Swiss Tropical and Public Health Institute**, Basel, Switzerland.

**Prof Angel Gil de Miguel**, Professor in the Department of Preventive Medicine and Public Health and Dean in Development and Cooperation, Volunteering and International Relations at Rey Juan Carlos University, Madrid, Spain.

One anonymous reviewer.
